# Synthesis, crystal structure and Hirshfeld surface analysis of 4′-cyano-[1,1′-biphen­yl]-4-yl 3-(benz­yloxy)benzoate

**DOI:** 10.1107/S2056989024008570

**Published:** 2024-09-12

**Authors:** M. Harish Kumar, M. Vinduvahini, H. C. Devarajegowda, H. T. Srinivasa, B. S. Palakshamurthy

**Affiliations:** a Department of Physics Yuvaraja's College University of Mysore,Mysore 570005 Karnaataka India; b Department of Physics, Maharani’s Science College for Women(Autonomous) Mysore, Karnataka, 750005, India; c Raman Research Institute, C V Raman Avenue Sadashivanagar Bangalore Karnataka India; d Department of PG Studies and Research in Physics Albert Einstein Block UCS Tumkur University, Tumkur Karnataka 572103 India; University of Aberdeen, United Kingdom

**Keywords:** crystal structure, Hirshfeld surface, 4′-cyano-[1,1′-biphen­yl], (benz­yloxy)benzoate

## Abstract

In the title compound, C_27_H_19_O_3_N, the dihedral angle between the aromatic rings of the biphenyl unit is 38.14 (2)° and the C—O—C—C torsion angle in the benz­yloxy benzene fragment is 179.1 (2)°. In the crystal, the mol­ecules are linked by weak C—H⋯O inter­actions forming *S*(9) chains propagating along [010]. The most important contributions to the Hirshfeld surface arise from H⋯H (32.4%) and C⋯H/H⋯C (37.0%) contacts.

## Abstract 2

The mol­ecules of the title compound are linked by weak C—H⋯O and C—H⋯π inter­actions.

## 1. Chemical context

Cyano­biphenyl-substituted derivatives can act as biological inhibitors and potential agents for the treatment of Alzheimer’s disease (Godyń *et al.*, 2021 ▸) as well as anti­bacterial and anti­malarial drugs (Malani *et al.*, 2013 ▸). Benz­yloxy derivatives exhibit anti-bacterial, anti-platelet and anti-malarial activities (Kaushik *et al.*, 2018 ▸; de Candia *et al.*, 2015 ▸; Mohebi *et al.*, 2022 ▸) and related pyrimidinyl­phenyl­amine derivatives are most active towards the inhibition of HIV-1 (Rai *et al.*, 2013 ▸). The cyano­biphenyl and (benz­yloxy)benzoate groups exhibit distinct structural geometries and these derivatives play significant roles in the construction of organic liquid crystal materials (Srinivasa *et al.*, 2015 ▸), which have been investigated for their display technology applications, such as optoelectronic materials, sensor materials, light-emitting diodes, and photovoltaic solar cells (Goodby *et al.*, 2022 ▸; Srinivasa *et al.*, 2024 ▸). As part of our studies of this family of materials, we now present the synthesis, structure and Hirshfeld surface analysis of the title compound, C_27_H_19_NO_3_ (I).
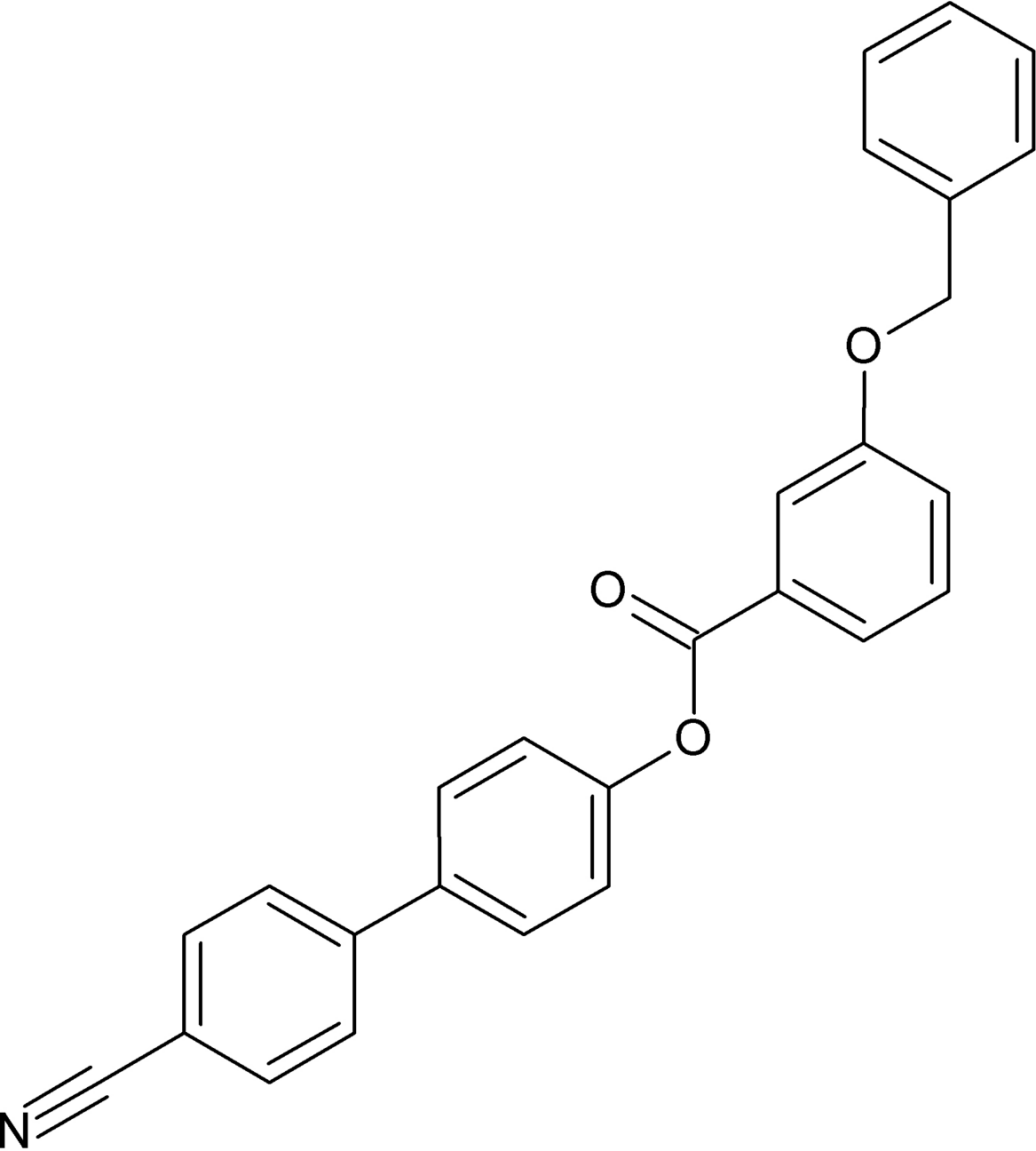


## 2. Structural commentary

**Figure 1:**
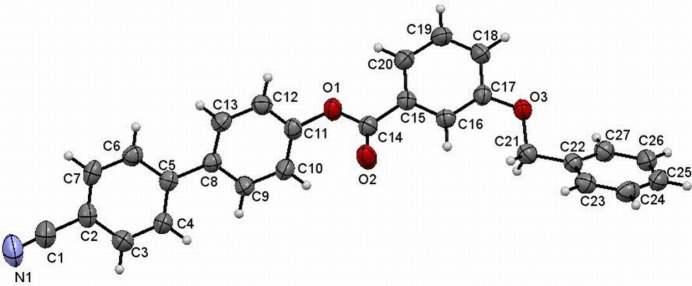
The mol­ecular structure of (I) with displacement ellipsoids drawn at the 50% probability level.

The mol­ecular structure of (I) is shown in Fig. 1 ▸. The aromatic rings in the mol­ecule are designated as *A* (C2–C7), *B* (C8–C13), *C* (C15–C20) and *D* (C22–C27) and the dihedral angles between the rings *A*/*B* = 38.14 (2), *A*/*C* = 8.29 (3) and *A*/*D* = 50.66 (2)°, whereas *B*/*C*, *B*/*D* and *C*/*D* are 46.43 (4), 83.95 (2) and 44.01 (2)°, respectively. The torsion angle associated with the phenyl benzoate group (C11—O1—C14—C15) is −177.8 (2)° and that for the benz­yloxy group (C22—C21—O3—C17) is 179.1 (2)°. Otherwise, the bond distances and angles may be regarded as normal.

## 3. Supra­molecular features

**Figure 2:**
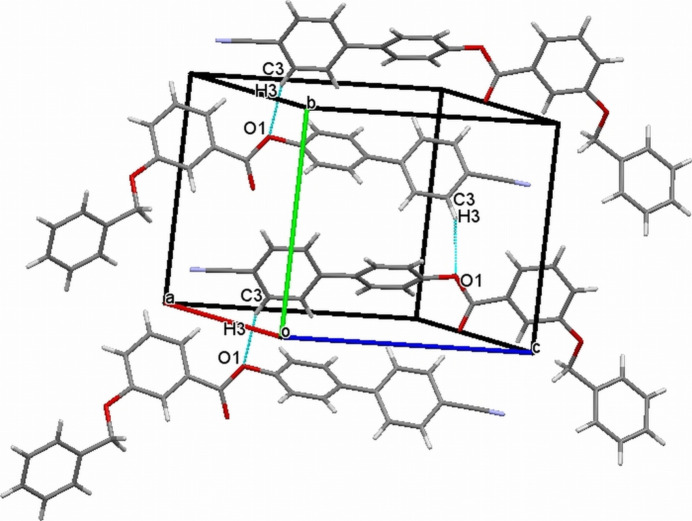
The crystal structure of (I) with a weak C—H ⋯ O inter­action forming an *S*(9) chain running along the [010] direction.

**Figure 3:**
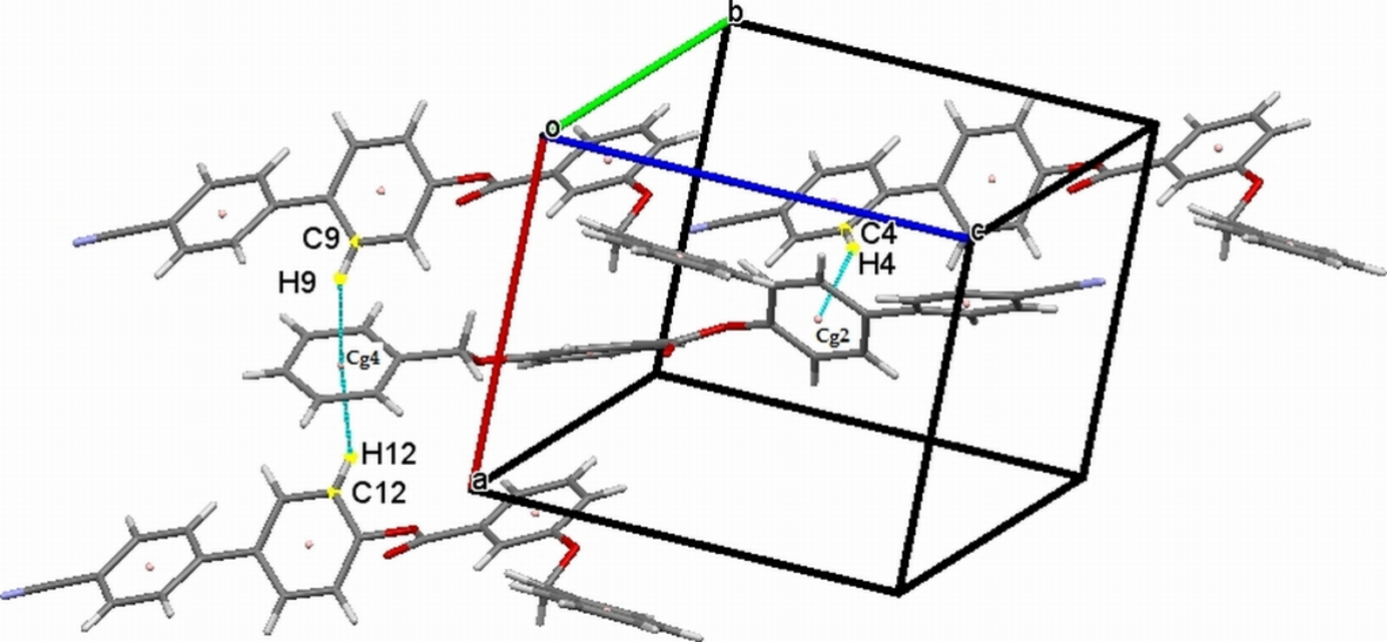
The mol­ecular packing of (I) with C—H⋯π inter­actions depicted by dashed lines.

**Figure 4:**
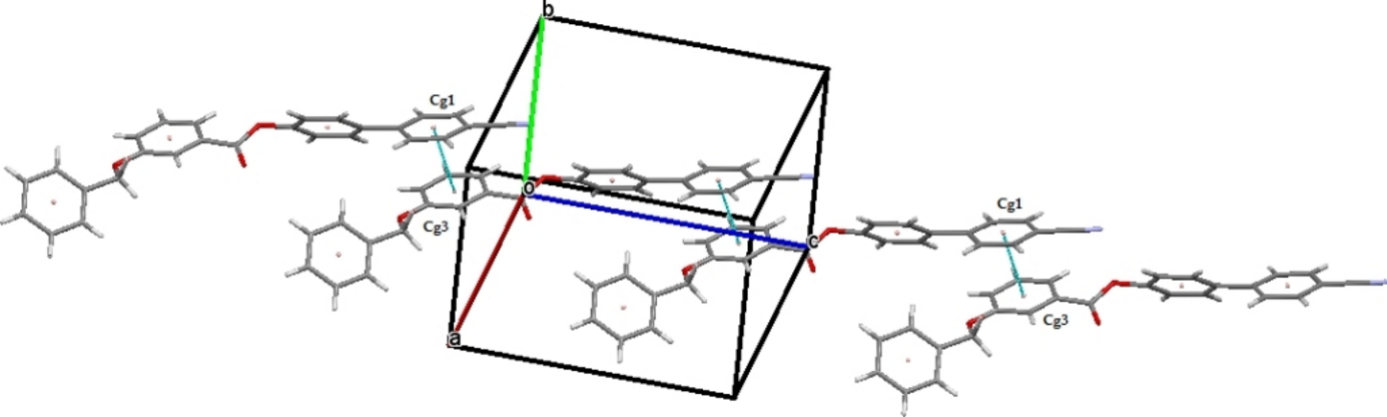
The mol­ecular packing of (I) with π–π inter­actions depicted by pale green coloured dashed lines.

The crystal structure features a weak C3—H3⋯O1 inter­action (Table 1 ▸), which forms an *S*(9) chain propagating along the [010] direction as shown in Fig. 2 ▸. Furthermore, the packing is consolidated by three weak C—H⋯π inter­actions as shown in Fig. 3 ▸. In addition there exists an aromatic π–π stacking inter­action between the C2–C7 and C15–C20 rings with a centroid–centroid distance of 3.9282 (19) Å (Fig. 4 ▸).

## 4. Hirshfeld surface analysis

**Figure 5:**
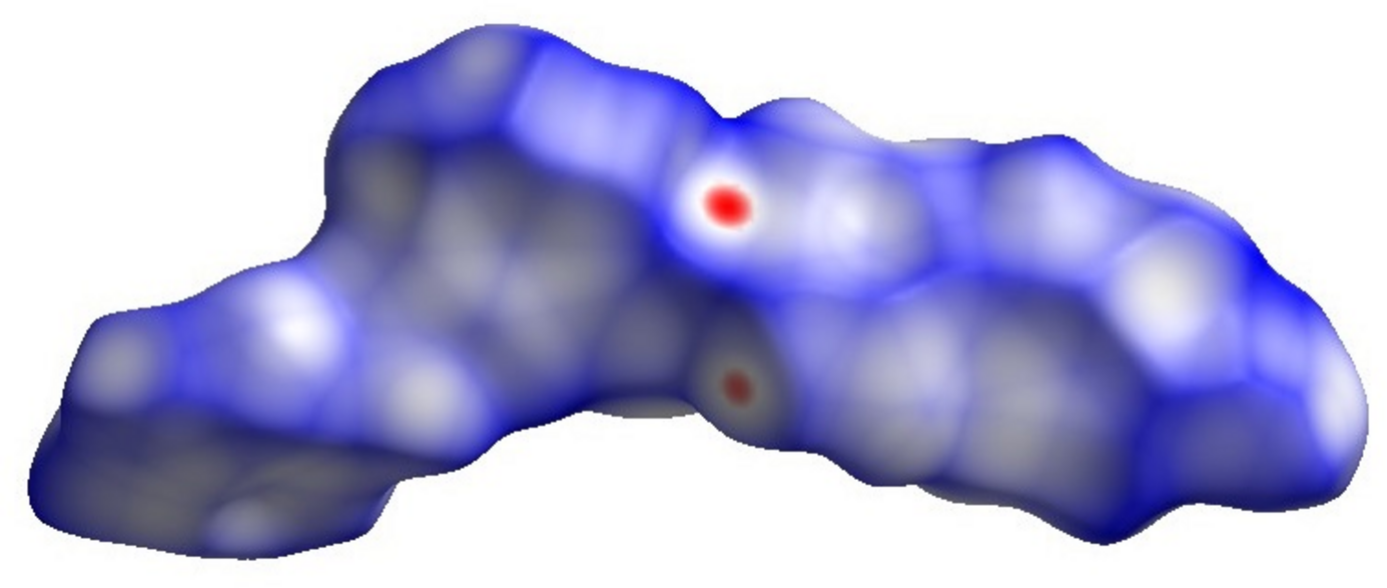
Hirshfeld surface representation for (I) plotted over *d*_norm_.

**Figure 6:**
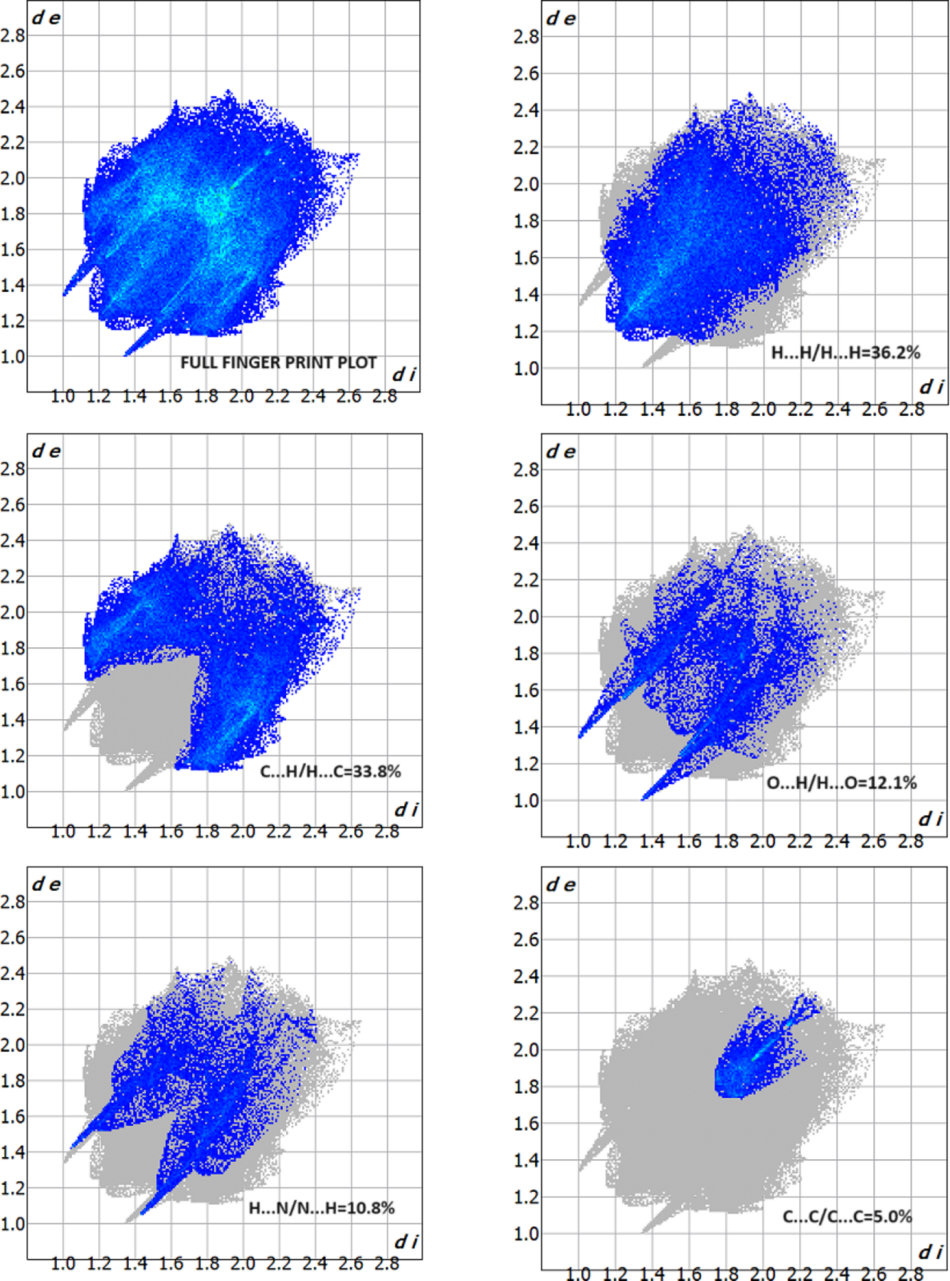
The full two-dimensional fingerprint plots for the title compound, showing all inter­actions and delineated into H⋯H, C⋯H/H⋯C, O⋯H/H⋯O, N⋯H/H⋯N and C⋯C inter­actions.

*CrystalExplorer17.5* (Turner *et al.*, 2017 ▸) was used to perform a Hirshfeld surface analysis to further qu­antify the various inter­molecular inter­actions. Fig. 5 ▸ illustrates the Hirshfeld surface mapped over *d*_norm_ with red spots corresponding to short contacts. The fingerprint plots (Fig. 6 ▸) indicate that the major contributions to the crystal structure are from H⋯H (36.2%), C⋯H/H⋯C (33.8%), O⋯H/H⋯O (12.1.6%), N⋯H/H⋯N (10.1.8%) and C⋯C (5.0%) contacts. The characteristic spikes in the O⋯H/H⋯O plot indicate the existence of the C—H⋯O hydrogen bond listed in Table 1 ▸.

## 5. Database survey

A search of the Cambridge Structural Database (CSD, version 5.42, update of November 2020; Groom *et al.*, 2016 ▸) for mol­ecules containing the 4′-cyano-[1,1′-biphen­yl] fragment resulted in two matches with CSD refcodes PIFZEN and PIFZIR (Jakubowski *et al.*, 2023 ▸). In these structures, the dihedral angle between the 4-cyano­phen­oxy ring and the neighbouring ring are 31.71 (2) and 38.95 (3)°, respectively, compared to 38.14 (2)° for (I). For mol­ecules containing the (benz­yloxy)benzoate fragment, a search resulted in thirteen matches: in all of these, the torsion angle of the linking C—O—C—C unit indicates a conformation close to *anti*.

## 6. Synthesis and crystallization

A mixture of 3-(benz­yloxy)benzoic acid (1 eq., 0.228 g) and 4′-hy­droxy-[1,1′-biphen­yl]-4-carbo­nitrile (1 eq., 0.195 g), di­cyclo­hexyl­carbodi­imide (1.2 eq.) and a catalytic amount of di­methyl­amino­pyrimidine were stirred in dry di­chloro­methane at room temperature overnight. After completion of the reaction, the product mass was subjected to column chromatography with silica gel and chloro­form as eluent. The crude product was recrystallized from chloro­form solution to yield colourless blocks of (I). Melting point = 398 K, analysis (%) calculated for C_27_H_19_NO_3_, C 79.98, H 4.72, N 3.45; found C 78.01; H 4.76, N 3.48. ^1^H NMR (500 MHz, CDCl_3_, δ/ppm): 7.82 (*m*, 4H, Ar-H), 7.65 (*m*, 4H, Ar-H), 7.44 (*m*, 4H, Ar-H), 7.23 (*m*, 5H, Ar-H), 5.24 (*s*, 2H, Ar—CH_2_—O—).

## 7. Refinement

Crystal data, data collection and structure refinement details are summarized in Table 2 ▸. All the H-atoms were positioned with idealized geometry and refined using a riding model with C—H = 0.93–0.97 Å and *U*_iso_(H) = 1.2*U*_eq_(C) or 1.5*U*_eq_(methyl C).

## Supplementary Material

Crystal structure: contains datablock(s) I. DOI: 10.1107/S2056989024008570/hb8105sup1.cif

Structure factors: contains datablock(s) I. DOI: 10.1107/S2056989024008570/hb8105Isup2.hkl

Supporting information file. DOI: 10.1107/S2056989024008570/hb8105Isup3.cml

CCDC reference: 2380701

Additional supporting information:  crystallographic information; 3D view; checkCIF report

## Figures and Tables

**Table 1:** Hydrogen-bond geometry (Å, °) *Cg*4 is the centroid of the C22–C27 ring.

*D*—H⋯*A*	*D*—H	H⋯*A*	*D*⋯*A*	*D*—H⋯*A*
C3—H3⋯O1^i^	0.93	2.52	3.395 (4)	158
CH—H4⋯*Cg*2^i^	0.93	2.97	3.805 (3)	151
C9—H9⋯*Cg*4^ii^	0.93	2.90	3.579 (3)	131
C12—H12⋯*Cg*4^iii^	0.93	2.77	3.485 (13)	135

Symmetry codes: (i) 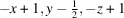; (ii) 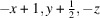; (iii) 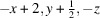.

**Table 2:** Experimental details

Crystal data	
Chemical formula	C_27_H_19_NO_3_
*M*_r_	405.43
Crystal system, space group	Monoclinic, *P*2_1_
Temperature (K)	270
*a*, *b*, *c* (Å)	9.4289 (10), 9.6739 (9), 11.4872 (11)
β (°)	97.668 (4)
*V* (Å^3^)	1038.43 (18)
*Z*	2
Radiation type	Mo *K*α
μ (mm^−1^)	0.09
Crystal size (mm)	0.42 × 0.38 × 0.24

Data collection	
Diffractometer	Bruker *SMART* APEXII CCD
Absorption correction	Multi-scan (*SADABS*; Krause *et al.*, 2015 ▸)
*T*_min_, *T*_max_	0.966, 0.981
No. of measured, independent and observed [*I* > 2σ(*I*)] reflections	27994, 3639, 3308
*R*_int_	0.052
(sin θ/λ)_max_ (Å^−1^)	0.595

Refinement	
*R*[*F*^2^ > 2σ(*F*^2^)], *wR*(*F*^2^), *S*	0.038, 0.080, 1.08
No. of reflections	3639
No. of parameters	280
No. of restraints	1
H-atom treatment	H-atom parameters constrained
Δρ_max_, Δρ_min_ (e Å^−3^)	0.11, −0.16
Absolute structure	Flack *x* determined using 1347 quotients [(*I*^+^)−(*I*^−^)]/[(*I*^+^)+(*I*^−^)] (Parsons *et al.*, 2013 ▸)
Absolute structure parameter	0.0 (5)

Computer programs: *APEX2* and *SAINT* (Bruker, 2014 ▸), *SHELXS97* (Sheldrick, 2008 ▸), *SHELXL2018/3* (Sheldrick, 2015 ▸) and *Mercury* (Macrae *et al.*, 2020 ▸).

## supplementary crystallographic information

### 4'-Cyano-[1,1'-biphenyl]-4-yl 3-(benzyloxy)benzoate Crystal data

**Table:**

C_27_H_19_NO_3_	Rod
*M**_r_* = 405.43	*D*_x_ = 1.297 Mg m^−^^3^
Monoclinic, *P*2_1_	Mo *K*α radiation, λ = 0.71073 Å
*a* = 9.4289 (10) Å	Cell parameters from 3639 reflections
*b* = 9.6739 (9) Å	θ = 3.5–25.1°
*c* = 11.4872 (11) Å	µ = 0.09 mm^−^^1^
β = 97.668 (4)°	*T* = 270 K
*V* = 1038.43 (18) Å^3^	Block, colourless
*Z* = 2	0.42 × 0.38 × 0.24 mm
*F*(000) = 424	

### 4'-Cyano-[1,1'-biphenyl]-4-yl 3-(benzyloxy)benzoate Data collection

**Table:**

Bruker SMART APEXII CCD diffractometer	3639 independent reflections
Radiation source: fine-focus sealed tube	3308 reflections with *I* > 2σ(*I*)
Graphite monochromator	*R*_int_ = 0.052
Detector resolution: 1.09 pixels mm^-1^	θ_max_ = 25.0°, θ_min_ = 3.6°
φ and Ω scans	*h* = −11→11
Absorption correction: multi-scan (SADABS; Krause *et al.*, 2015)	*k* = −11→11
*T*_min_ = 0.966, *T*_max_ = 0.981	*l* = −13→13
27994 measured reflections	

### 4'-Cyano-[1,1'-biphenyl]-4-yl 3-(benzyloxy)benzoate Refinement

**Table:**

Refinement on *F*^2^	Secondary atom site location: difference Fourier map
Least-squares matrix: full	Hydrogen site location: inferred from neighbouring sites
*R*[*F*^2^ > 2σ(*F*^2^)] = 0.038	H-atom parameters constrained
*wR*(*F*^2^) = 0.080	*w* = 1/[σ^2^(*F*_o_^2^) + (0.0339*P*)^2^ + 0.1198*P*] where *P* = (*F*_o_^2^ + 2*F*_c_^2^)/3
*S* = 1.08	(Δ/σ)_max_ < 0.001
3639 reflections	Δρ_max_ = 0.11 e Å^−^^3^
280 parameters	Δρ_min_ = −0.16 e Å^−^^3^
1 restraint	Absolute structure: Flack *x* determined using 1347 quotients [(*I*^+^)-(*I*^-^)]/[(*I*^+^)+(*I*^-^)] (Parsons *et al.*, 2013)
0.012 constraints	Absolute structure parameter: 0.0 (5)
Primary atom site location: structure-invariant direct methods	

### 4'-Cyano-[1,1'-biphenyl]-4-yl 3-(benzyloxy)benzoate Special details

**Table:**

Geometry. All esds (except the esd in the dihedral angle between two l.s. planes) are estimated using the full covariance matrix. The cell esds are taken into account individually in the estimation of esds in distances, angles and torsion angles; correlations between esds in cell parameters are only used when they are defined by crystal symmetry. An approximate (isotropic) treatment of cell esds is used for estimating esds involving l.s. planes.

### 4'-Cyano-[1,1'-biphenyl]-4-yl 3-(benzyloxy)benzoate Fractional atomic coordinates and isotropic or equivalent isotropic displacement parameters (Å^2^)

**Table:**

	*x*	*y*	*z*	*U*_iso_*/*U*_eq_	
O1	0.7334 (2)	0.77894 (19)	0.21126 (16)	0.0509 (5)	
O3	0.8888 (2)	0.5498 (2)	−0.24091 (16)	0.0555 (6)	
O2	0.7452 (3)	0.5514 (2)	0.17589 (19)	0.0695 (7)	
C11	0.6854 (3)	0.7566 (3)	0.3203 (2)	0.0430 (7)	
C13	0.7200 (3)	0.7988 (3)	0.5258 (2)	0.0443 (7)	
H13	0.775740	0.834123	0.592030	0.053*	
C21	0.8166 (3)	0.4201 (3)	−0.2372 (2)	0.0484 (7)	
H21A	0.715024	0.435077	−0.236419	0.058*	
H21B	0.853943	0.369863	−0.166652	0.058*	
C10	0.5549 (3)	0.6967 (3)	0.3284 (2)	0.0506 (7)	
H10	0.498693	0.663636	0.261571	0.061*	
C16	0.8197 (3)	0.6095 (3)	−0.0507 (2)	0.0414 (6)	
H16	0.783496	0.521562	−0.040255	0.050*	
C8	0.5898 (3)	0.7372 (3)	0.5372 (2)	0.0384 (6)	
C22	0.8404 (3)	0.3390 (3)	−0.3434 (2)	0.0401 (6)	
C12	0.7683 (3)	0.8085 (3)	0.4174 (2)	0.0450 (7)	
H12	0.855882	0.849784	0.410635	0.054*	
C20	0.8662 (3)	0.8422 (3)	0.0195 (2)	0.0488 (7)	
H20	0.860968	0.910130	0.076078	0.059*	
C27	0.8103 (3)	0.3941 (3)	−0.4553 (3)	0.0481 (7)	
H27	0.775214	0.483768	−0.465142	0.058*	
C17	0.8782 (3)	0.6410 (3)	−0.1512 (2)	0.0421 (6)	
C9	0.5083 (3)	0.6864 (3)	0.4366 (2)	0.0470 (7)	
H9	0.420768	0.644553	0.442524	0.056*	
C15	0.8154 (3)	0.7109 (3)	0.0354 (2)	0.0391 (6)	
C6	0.5598 (3)	0.8325 (3)	0.7351 (2)	0.0461 (7)	
H6	0.611277	0.910110	0.717919	0.055*	
C5	0.5377 (3)	0.7261 (3)	0.6536 (2)	0.0393 (6)	
C2	0.4316 (3)	0.7098 (3)	0.8675 (2)	0.0493 (7)	
C4	0.4646 (3)	0.6095 (3)	0.6828 (2)	0.0496 (7)	
H4	0.451143	0.536161	0.630193	0.060*	
C19	0.9250 (3)	0.8715 (3)	−0.0818 (3)	0.0550 (8)	
H19	0.959121	0.959983	−0.093327	0.066*	
C23	0.8922 (3)	0.2051 (3)	−0.3313 (3)	0.0475 (7)	
H23	0.911977	0.166328	−0.256862	0.057*	
N1	0.3247 (4)	0.6894 (4)	1.0630 (3)	0.0907 (11)	
C7	0.5067 (3)	0.8251 (3)	0.8410 (2)	0.0506 (7)	
H7	0.521498	0.897631	0.894393	0.061*	
C3	0.4113 (3)	0.6006 (3)	0.7889 (3)	0.0552 (8)	
H3	0.362120	0.521913	0.807373	0.066*	
C14	0.7605 (3)	0.6679 (3)	0.1449 (3)	0.0450 (7)	
C18	0.9338 (3)	0.7717 (3)	−0.1657 (2)	0.0515 (8)	
H18	0.976819	0.791722	−0.232010	0.062*	
C26	0.8322 (3)	0.3164 (3)	−0.5526 (3)	0.0549 (8)	
H26	0.811223	0.353879	−0.627419	0.066*	
C25	0.8848 (3)	0.1843 (3)	−0.5389 (3)	0.0588 (8)	
H25	0.900050	0.132486	−0.604287	0.071*	
C1	0.3728 (4)	0.6998 (4)	0.9774 (3)	0.0635 (9)	
C24	0.9147 (3)	0.1288 (3)	−0.4283 (3)	0.0561 (8)	
H24	0.950410	0.039321	−0.418934	0.067*	

### 4'-Cyano-[1,1'-biphenyl]-4-yl 3-(benzyloxy)benzoate Atomic displacement parameters (Å^2^)

**Table:**

	*U*^11^	*U*^22^	*U*^33^	*U*^12^	*U*^13^	*U*^23^
O1	0.0725 (14)	0.0391 (10)	0.0449 (11)	0.0014 (10)	0.0219 (10)	−0.0050 (9)
O3	0.0780 (15)	0.0486 (12)	0.0448 (11)	−0.0129 (11)	0.0262 (10)	−0.0078 (9)
O2	0.112 (2)	0.0427 (12)	0.0623 (14)	−0.0043 (13)	0.0450 (13)	−0.0047 (11)
C11	0.0543 (17)	0.0372 (15)	0.0398 (15)	0.0058 (13)	0.0142 (13)	−0.0018 (12)
C13	0.0456 (16)	0.0440 (15)	0.0421 (15)	−0.0021 (13)	0.0016 (12)	−0.0031 (12)
C21	0.0514 (17)	0.0455 (16)	0.0506 (17)	−0.0032 (13)	0.0152 (14)	−0.0025 (14)
C10	0.0530 (17)	0.0555 (18)	0.0437 (16)	−0.0063 (15)	0.0079 (13)	−0.0136 (14)
C16	0.0458 (15)	0.0361 (14)	0.0431 (15)	−0.0021 (12)	0.0085 (12)	0.0004 (12)
C8	0.0385 (14)	0.0334 (14)	0.0435 (15)	0.0041 (11)	0.0066 (11)	−0.0026 (11)
C22	0.0382 (14)	0.0404 (15)	0.0432 (16)	−0.0017 (12)	0.0103 (12)	−0.0023 (12)
C12	0.0445 (15)	0.0408 (15)	0.0508 (17)	−0.0009 (13)	0.0110 (13)	−0.0008 (13)
C20	0.0572 (17)	0.0453 (17)	0.0435 (16)	−0.0077 (14)	0.0050 (13)	−0.0047 (13)
C27	0.0515 (17)	0.0429 (16)	0.0514 (18)	0.0016 (13)	0.0130 (14)	−0.0007 (13)
C17	0.0487 (15)	0.0428 (15)	0.0350 (15)	−0.0007 (13)	0.0060 (12)	−0.0027 (12)
C9	0.0429 (15)	0.0548 (17)	0.0445 (16)	−0.0065 (14)	0.0102 (12)	−0.0083 (14)
C15	0.0385 (14)	0.0403 (15)	0.0386 (14)	0.0012 (12)	0.0048 (11)	−0.0003 (12)
C6	0.0544 (17)	0.0361 (15)	0.0479 (17)	0.0009 (13)	0.0068 (13)	−0.0029 (13)
C5	0.0402 (14)	0.0386 (14)	0.0387 (14)	0.0057 (12)	0.0033 (11)	−0.0012 (12)
C2	0.0559 (18)	0.0544 (18)	0.0377 (15)	0.0068 (15)	0.0067 (13)	−0.0004 (15)
C4	0.0586 (18)	0.0489 (17)	0.0427 (16)	−0.0082 (14)	0.0119 (13)	−0.0097 (13)
C19	0.071 (2)	0.0476 (17)	0.0465 (17)	−0.0191 (16)	0.0078 (15)	−0.0018 (14)
C23	0.0473 (16)	0.0450 (16)	0.0511 (16)	−0.0004 (14)	0.0091 (13)	0.0027 (14)
N1	0.114 (3)	0.112 (3)	0.0516 (18)	0.004 (2)	0.0303 (18)	−0.0054 (19)
C7	0.0602 (18)	0.0490 (17)	0.0418 (16)	0.0078 (15)	0.0039 (14)	−0.0123 (13)
C3	0.063 (2)	0.0538 (17)	0.0510 (17)	−0.0105 (16)	0.0151 (15)	−0.0012 (14)
C14	0.0506 (16)	0.0398 (15)	0.0465 (16)	−0.0002 (13)	0.0131 (13)	−0.0048 (13)
C18	0.0608 (19)	0.0557 (17)	0.0390 (16)	−0.0142 (15)	0.0105 (14)	0.0036 (15)
C26	0.0610 (19)	0.062 (2)	0.0428 (17)	−0.0149 (16)	0.0112 (14)	−0.0030 (15)
C25	0.061 (2)	0.056 (2)	0.063 (2)	−0.0137 (17)	0.0226 (16)	−0.0223 (17)
C1	0.074 (2)	0.070 (2)	0.0475 (19)	0.0047 (19)	0.0114 (16)	−0.0063 (18)
C24	0.0561 (19)	0.0386 (16)	0.076 (2)	−0.0020 (14)	0.0166 (16)	−0.0101 (16)

### 4'-Cyano-[1,1'-biphenyl]-4-yl 3-(benzyloxy)benzoate Geometric parameters (Å, º)

**Table:**

O1—C14	1.361 (3)	C27—H27	0.9300
O1—C11	1.405 (3)	C17—C18	1.387 (4)
O3—C17	1.370 (3)	C9—H9	0.9300
O3—C21	1.431 (3)	C15—C14	1.482 (4)
O2—C14	1.197 (3)	C6—C7	1.378 (4)
C11—C12	1.369 (4)	C6—C5	1.388 (4)
C11—C10	1.374 (4)	C6—H6	0.9300
C13—C12	1.386 (4)	C5—C4	1.386 (4)
C13—C8	1.386 (4)	C2—C7	1.377 (4)
C13—H13	0.9300	C2—C3	1.386 (4)
C21—C22	1.492 (4)	C2—C1	1.448 (4)
C21—H21A	0.9700	C4—C3	1.381 (4)
C21—H21B	0.9700	C4—H4	0.9300
C10—C9	1.376 (4)	C19—C18	1.374 (4)
C10—H10	0.9300	C19—H19	0.9300
C16—C17	1.379 (4)	C23—C24	1.376 (4)
C16—C15	1.398 (4)	C23—H23	0.9300
C16—H16	0.9300	N1—C1	1.141 (4)
C8—C9	1.389 (4)	C7—H7	0.9300
C8—C5	1.488 (3)	C3—H3	0.9300
C22—C27	1.385 (4)	C18—H18	0.9300
C22—C23	1.385 (4)	C26—C25	1.372 (5)
C12—H12	0.9300	C26—H26	0.9300
C20—C15	1.378 (4)	C25—C24	1.373 (4)
C20—C19	1.384 (4)	C25—H25	0.9300
C20—H20	0.9300	C24—H24	0.9300
C27—C26	1.385 (4)		
			
C14—O1—C11	119.0 (2)	C20—C15—C14	122.5 (2)
C17—O3—C21	117.4 (2)	C16—C15—C14	116.8 (2)
C12—C11—C10	121.2 (2)	C7—C6—C5	121.2 (3)
C12—C11—O1	117.0 (3)	C7—C6—H6	119.4
C10—C11—O1	121.6 (2)	C5—C6—H6	119.4
C12—C13—C8	121.0 (3)	C4—C5—C6	118.3 (2)
C12—C13—H13	119.5	C4—C5—C8	120.7 (2)
C8—C13—H13	119.5	C6—C5—C8	121.0 (2)
O3—C21—C22	108.2 (2)	C7—C2—C3	120.3 (3)
O3—C21—H21A	110.1	C7—C2—C1	120.9 (3)
C22—C21—H21A	110.1	C3—C2—C1	118.8 (3)
O3—C21—H21B	110.1	C3—C4—C5	121.1 (3)
C22—C21—H21B	110.1	C3—C4—H4	119.5
H21A—C21—H21B	108.4	C5—C4—H4	119.5
C11—C10—C9	119.2 (3)	C18—C19—C20	120.9 (3)
C11—C10—H10	120.4	C18—C19—H19	119.5
C9—C10—H10	120.4	C20—C19—H19	119.5
C17—C16—C15	119.4 (2)	C24—C23—C22	120.7 (3)
C17—C16—H16	120.3	C24—C23—H23	119.6
C15—C16—H16	120.3	C22—C23—H23	119.6
C13—C8—C9	118.2 (2)	C2—C7—C6	119.7 (3)
C13—C8—C5	121.2 (2)	C2—C7—H7	120.2
C9—C8—C5	120.6 (2)	C6—C7—H7	120.2
C27—C22—C23	118.6 (3)	C4—C3—C2	119.5 (3)
C27—C22—C21	121.5 (3)	C4—C3—H3	120.3
C23—C22—C21	119.9 (3)	C2—C3—H3	120.3
C11—C12—C13	119.1 (3)	O2—C14—O1	122.5 (3)
C11—C12—H12	120.4	O2—C14—C15	125.9 (3)
C13—C12—H12	120.4	O1—C14—C15	111.5 (2)
C15—C20—C19	119.1 (3)	C19—C18—C17	119.8 (3)
C15—C20—H20	120.5	C19—C18—H18	120.1
C19—C20—H20	120.5	C17—C18—H18	120.1
C22—C27—C26	120.4 (3)	C25—C26—C27	120.3 (3)
C22—C27—H27	119.8	C25—C26—H26	119.9
C26—C27—H27	119.8	C27—C26—H26	119.9
O3—C17—C16	124.4 (2)	C26—C25—C24	119.8 (3)
O3—C17—C18	115.5 (2)	C26—C25—H25	120.1
C16—C17—C18	120.1 (2)	C24—C25—H25	120.1
C10—C9—C8	121.2 (3)	N1—C1—C2	178.4 (4)
C10—C9—H9	119.4	C25—C24—C23	120.3 (3)
C8—C9—H9	119.4	C25—C24—H24	119.9
C20—C15—C16	120.6 (2)	C23—C24—H24	119.9
			
C14—O1—C11—C12	120.6 (3)	C9—C8—C5—C4	37.8 (4)
C14—O1—C11—C10	−65.3 (4)	C13—C8—C5—C6	38.2 (4)
C17—O3—C21—C22	179.1 (2)	C9—C8—C5—C6	−141.5 (3)
C12—C11—C10—C9	−1.4 (4)	C6—C5—C4—C3	1.8 (4)
O1—C11—C10—C9	−175.3 (3)	C8—C5—C4—C3	−177.6 (3)
C12—C13—C8—C9	−0.5 (4)	C15—C20—C19—C18	−0.3 (5)
C12—C13—C8—C5	179.8 (2)	C27—C22—C23—C24	0.7 (4)
O3—C21—C22—C27	−52.9 (3)	C21—C22—C23—C24	180.0 (3)
O3—C21—C22—C23	127.8 (3)	C3—C2—C7—C6	1.0 (5)
C10—C11—C12—C13	0.8 (4)	C1—C2—C7—C6	−179.1 (3)
O1—C11—C12—C13	174.9 (2)	C5—C6—C7—C2	0.7 (4)
C8—C13—C12—C11	0.2 (4)	C5—C4—C3—C2	−0.2 (4)
C23—C22—C27—C26	−0.2 (4)	C7—C2—C3—C4	−1.2 (5)
C21—C22—C27—C26	−179.5 (3)	C1—C2—C3—C4	178.9 (3)
C21—O3—C17—C16	9.1 (4)	C11—O1—C14—O2	0.0 (4)
C21—O3—C17—C18	−172.0 (3)	C11—O1—C14—C15	−177.8 (2)
C15—C16—C17—O3	179.6 (3)	C20—C15—C14—O2	−162.2 (3)
C15—C16—C17—C18	0.8 (4)	C16—C15—C14—O2	14.9 (4)
C11—C10—C9—C8	1.0 (4)	C20—C15—C14—O1	15.5 (4)
C13—C8—C9—C10	−0.1 (4)	C16—C15—C14—O1	−167.4 (2)
C5—C8—C9—C10	179.6 (3)	C20—C19—C18—C17	2.4 (5)
C19—C20—C15—C16	−1.6 (4)	O3—C17—C18—C19	178.4 (3)
C19—C20—C15—C14	175.4 (3)	C16—C17—C18—C19	−2.7 (4)
C17—C16—C15—C20	1.3 (4)	C22—C27—C26—C25	−0.4 (4)
C17—C16—C15—C14	−175.9 (2)	C27—C26—C25—C24	0.5 (4)
C7—C6—C5—C4	−2.0 (4)	C26—C25—C24—C23	0.0 (4)
C7—C6—C5—C8	177.3 (3)	C22—C23—C24—C25	−0.6 (4)
C13—C8—C5—C4	−142.5 (3)		

### 4'-Cyano-[1,1'-biphenyl]-4-yl 3-(benzyloxy)benzoate Hydrogen-bond geometry (Å, º)

*Cg*4 is the centroid of the C22–C27 ring.

**Table:**

*D*—H···*A*	*D*—H	H···*A*	*D*···*A*	*D*—H···*A*
C3—H3···O1^i^	0.93	2.52	3.395 (4)	158
CH—H4···*Cg*2^i^	0.93	2.97	3.805 (3)	151
C9—H9···*Cg*4^ii^	0.93	2.90	3.579 (3)	131
C12—H12···*Cg*4^iii^	0.93	2.77	3.485 (13)	135

Symmetry codes: (i) −*x*+1, *y*−1/2, −*z*+1; (ii) −*x*+1, *y*+1/2, −*z*; (iii) −*x*+2, *y*+1/2, −*z*.

## References

[bb1] Bruker (2014). *APEX3* and *SAINT*. Bruker AXS Inc., Madison, Wisconsin, USA.

[bb2] Candia, M. de, Marini, E., Zaetta, G., Cellamare, S., Di Stilo, A. & Altomare, C. D. (2015). *Eur. J. Pharm. Sci.* **72**, 69–80. 10.1016/j.ejps.2015.03.004 25769522

[bb3] Godyń, J., Zaręba, P., Łażewska, D., Stary, D., Reiner-Link, D., Frank, A., Latacz, G., Mogilski, S., Kaleta, M., Doroz-Płonka, A., Lubelska, A., Honkisz-Orzechowska, E., Olejarz-Maciej, A., Handzlik, J., Stark, H., Kieć-Kononowicz, K., Malawska, B. & Bajda, M. (2021). *Bioorg. Chem.* **114**, 105129. 10.1016/j.bioorg.2021.105129 34217977

[bb4] Goodby, J. W., Cowling, S. J., Bradbury, C. K. & Mandle, R. J. (2022). *Liq. Cryst.* **49**, 908–933.

[bb5] Groom, C. R., Bruno, I. J., Lightfoot, M. P. & Ward, S. C. (2016). *Acta Cryst.* B**72**, 171–179. 10.1107/S2052520616003954 PMC4822653 27048719

[bb6] Jakubowski, R., Januszko, A., William Tilford, R., Radziszewski, G. J., Pietrzak, A., Young, V. G. Jr & Kaszyński, P. (2023). *Chem. A Eur. J.* **29**, e202203948. 10.1002/chem.202203948 36813741

[bb7] Kaushik, C. P., Pahwa, A., Kumar, D., Kumar, A., Singh, D., Kumar, K. & Luxmi, R. (2018). *J. Heterocycl. Chem.* **55**, 1720–1728.

[bb8] Krause, L., Herbst-Irmer, R., Sheldrick, G. M. & Stalke, D. (2015). *J. Appl. Cryst.* **48**, 3–10. 10.1107/S1600576714022985 PMC4453166 26089746

[bb9] Macrae, C. F., Sovago, I., Cottrell, S. J., Galek, P. T. A., McCabe, P., Pidcock, E., Platings, M., Shields, G. P., Stevens, J. S., Towler, M. & Wood, P. A. (2020). *J. Appl. Cryst.* **53**, 226–235. 10.1107/S1600576719014092 PMC6998782 32047413

[bb10] Malani, M. H. & Dholakiya, B. Z. (2013). *Bioorg. Chem.* **51**, 16–23. 10.1016/j.bioorg.2013.09.001 24080364

[bb11] Mohebi, M., Fayazi, N., Esmaeili, S., Rostami, M., Bagheri, F., Aliabadi, A., Asadi, P. & Saghaie, L. (2022). *Res Pharma Sci*, **17**, 252–264. 10.4103/1735-5362.343079 PMC9075022 35531137

[bb12] Parsons, S., Flack, H. D. & Wagner, T. (2013). *Acta Cryst.* B**69**, 249–259. 10.1107/S2052519213010014 PMC3661305 23719469

[bb13] Rai, D., Chen, W., Tian, Y., Chen, X., Zhan, P., De Clercq, E., Pannecouque, C., Balzarini, J. & Liu, X. (2013). *Bioorg. Med. Chem.* **21**, 7398–7405. 10.1016/j.bmc.2013.09.051 24134904

[bb14] Sheldrick, G. M. (2008). *Acta Cryst.* A**64**, 112–122. 10.1107/S0108767307043930 18156677

[bb15] Sheldrick, G. M. (2015). *Acta Cryst.* C**71**, 3–8.

[bb16] Srinivasa, H. T. & Hariprasad, S. (2024). *Phase Transit.* **97**, 201–211.

[bb17] Srinivasa, H. T., Palakshamurthy, B. S., Velmurugan, D., Devarajegowda, H. C. & Hariprasad, S. (2015). *Acta Chim. Slov.* **62**, 768–774.

[bb18] Turner, M. J., MacKinnon, J. J., Wolff, S. K., Grimwood, D. J., Spackman, P. R., Jayatilaka, D. & Spackman, M. A. (2017). *CrystalExplorer17.5.* University of Western Australia. http:// hirshfeldsurface. net.

